# White‐crowned sparrow males show immediate flexibility in song amplitude but not in song minimum frequency in response to changes in noise levels in the field

**DOI:** 10.1002/ece3.3037

**Published:** 2017-05-31

**Authors:** Elizabeth P. Derryberry, Katherine Gentry, Graham E. Derryberry, Jennifer N. Phillips, Raymond M. Danner, Julie E. Danner, David A. Luther

**Affiliations:** ^1^ Department of Ecology and Evolutionary BiologyTulane UniversityNew OrleansLAUSA; ^2^ Biology DepartmentGeorge Mason UniversityFairfaxVAUSA; ^3^ Museum of Natural ScienceLouisiana State UniversityBaton RougeLAUSA; ^4^ Present address: Department of Biology and Marine BiologyUniversity of North CarolinaWilmingtonNCUSA

**Keywords:** anthropogenic noise, behavioral plasticity, birdsong, cultural evolution, soundscape

## Abstract

The soundscape acts as a selective agent on organisms that use acoustic signals to communicate. A number of studies document variation in structure, amplitude, or timing of signal production in correspondence with environmental noise levels thus supporting the hypothesis that organisms are changing their signaling behaviors to avoid masking. The time scale at which organisms respond is of particular interest. Signal structure may evolve across generations through processes such as cultural or genetic transmission. Individuals may also change their behavior during development (ontogenetic change) or in real time (i.e., immediate flexibility). These are not mutually exclusive mechanisms, and all must be investigated to understand how organisms respond to selection pressures from the soundscape. Previous work on white‐crowned sparrows (*Zonotrichia leucophrys*) found that males holding territories in louder areas tend to sing higher frequency songs and that both noise levels and song frequency have increased over time (30 years) in urban areas. These previous findings suggest that songs are changing across generations; however, it is not known if this species also exhibits immediate flexibility. Here, we conducted an exploratory, observational study to ask whether males change the minimum frequency of their song in response to immediate changes in noise levels. We also ask whether males sing louder, as increased minimum frequency may be physiologically linked to producing sound at higher amplitudes, in response to immediate changes in environmental noise. We found that territorial males adjust song amplitude but not minimum frequency in response to changes in environmental noise levels. Our results suggest that males do not show immediate flexibility in song minimum frequency, although experimental manipulations are needed to test this hypothesis further. Our work highlights the need to investigate multiple mechanisms of adaptive response to soundscapes.

## 1 INTRODUCTION

Selection acts on organisms to maximize the transfer of information from signaler to an intended receiver during communication (Endler, 1993; Morton, 1975). Thus, signals, signaling behaviors, and receptors are expected to vary with respect to the environment in which communication is taking place (Endler, 1993, 2000; Wiley, 2006, 2013). Many aspects of the environment constrain acoustic communication, including vegetation structure (Wiley & Richards, 1978), microclimate conditions (Wiley & Richards, 1982), and background noise levels (Klump, 1996). When background noise and signal frequencies overlap, background noise levels can mask the signal, reducing the distance at which a receiver can detect and discriminate information (Klump, 1996). Therefore, the sounds that make up a landscape—the soundscape (Pijanowski et al., 2011)—can act as a selective agent on acoustic signals. There has been recent recognition of the evolutionarily unprecedented increase in noise pollution in many soundscapes due to human activity (Swaddle et al., 2015). A pressing question then, both from a basic ecological perspective (Francis, Kleist, Ortega, & Cruz, 2012) as well as an applied perspective (Barber, Crooks, & Fristrup, 2010; Francis & Barber, 2013), is how are organisms responding (or not) to changes in their soundscape.

Acoustic signals vary with environmental noise levels, suggesting that signal structure evolves over generations in response to selective pressures from soundscapes. Both bird and frog species living in very high noise environments—such as next to waterfalls—produce narrower bandwidth vocalizations with greater internote intervals, presumably with a reduction in masking by the sound of rushing water (Dubois & Martens, 1984). Populations within a species may also diverge in signal phenotype due to occupation of different soundscapes. For example, Slabbekoorn and Smith (2002) found that the songs of little greenbuls (*Andropadus virens*) diverged in spectral traits between populations occupying vegetation types with different background noise spectrums due to insect noise (the frequency distribution of sound level). Similarly, populations of two species of frog in Australia found near roads produce calls at a higher pitch than populations not exposed to traffic noise (Parris, Velik‐Lord, & North, 2009). Changes in environmental noise levels over time have also been implicated in the evolution of spectral traits of songs within a bird population (Luther & Baptista, 2010; Luther & Derryberry, 2012). Thus, vocal signals appear to evolve in ways that maximize the signal‐to‐noise ratio and reduce masking in response to variation in soundscapes.

Another evolutionary response is the degree of behavioral plasticity in singing behaviors (Brumm & Zollinger, 2011; Slabbekoorn, 2013). Soundscapes can change within a generation, vary dramatically from day to day and even moment to moment (Pijanowski et al., 2011), and individuals better able to change their behavior in response are expected to have more successful communication (Patricelli & Blickley, 2006). This plastic response can occur during development (an ontogenetic response), for example, through selective attrition of masked signals or passive acquisition of signals that transmit well, or through short‐term adjustments (immediate flexibility) (Patricelli & Blickley, 2006). There is experimental evidence for ontogenetic adjustments in response to the environment (e.g., Nelson, D. A., 2000; Peters, Derryberry, & Nowicki, 2012), although none that we know of in the context of masking by environmental noise. Immediate flexibility in vocal behavior is taxonomically widespread. Echolocating bats adjust both call frequency and amplitude in response to changes in ambient noise levels (Hage, Jiang, Berquist, Feng, & Metzner, 2013). Killer whales compensate for vessel noise by increasing call amplitude (Holt, Noren, Veirs, Emmons, & Veirs, 2009). Many bird species also sing at higher song amplitude, higher pitch, and/or longer songs with increasing levels of traffic noise to reduce masking (reviewed in Brumm & Zollinger, 2013). In some bird species, individuals will sing with either greater redundancy (repetition of songs or notes, for example, of introductory notes (Ríos‐Chelén, Quirós‐Guerrero, Gil, & Macías Garcia, 2012)) or at a slower rate, to increase the probability of transmitting information despite an increase in masking (Brumm & Zollinger, 2013). Immediate flexibility has been examined most extensively in birds, and in this taxonomic group some species change only one feature of their song or singing behavior, whereas others show multiple forms of immediate flexibility (reviewed in Brumm & Zollinger, 2013; Slabbekoorn, 2013). Thus, species vary in the degree to which they are flexible in their vocal behavior.

As detailed above, soundscapes probably drive changes in vocalizations over generations, either through genetic change or ontogenetic adjustments (i.e., cultural evolution), as well as the degree of immediate flexibility. However, we do not know the relative role of these types of evolutionary responses in explaining current variation in vocal behavior. More work is needed on these mechanisms, particularly in the same species. For example, Great Tits (*Parus major*) in urban areas produce song types with higher minimum frequencies than in rural areas (Slabbekoorn & Den Boer‐Visser, 2006; Slabbekoorn & Peet, 2003). One explanation for this spatial variation is that individuals are known to shift to song types with higher minimum frequencies with increases in noise levels (Halfwerk & Slabbekoorn, 2009). However, it is not known whether song structure, or the frequency of occurrence of different song types (Slabbekoorn, 2013), is changing over time in urban versus rural populations. Dark‐eyed Juncos (*Junco hyemalis*) also show spatial variation in song minimum frequency (Slabbekoorn, Yeh, & Hunt, 2007), and there is some suggestion that immediate flexibility is not sufficient to explain divergence in song between urban and rural populations, although both processes were assessed indirectly (Cardoso & Atwell, 2010). All three mechanisms are probably at work in many species, and yet we do not know the extent to which current signal variation reflects changes in the vocal phenotype over generations through evolution or developmental modification or is primarily a real‐time response to changes in noise levels (i.e., immediate flexibility).

**Figure 1:**
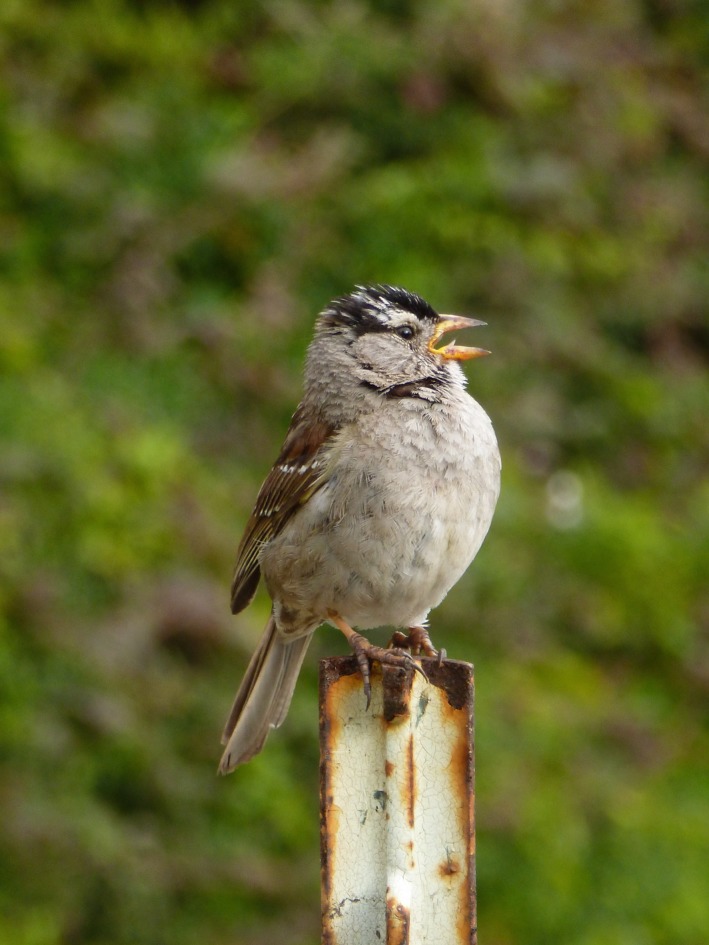
Male white‐crowned sparrow singing on his territory in San Francisco, CA. Photo credit: Jennifer N. Phillips

Here, we observed the singing behavior of free‐living, male Nuttall's white‐crowned sparrows (*Zonotrichia leucophrys nuttalli*) in the San Francisco, CA area (Figure 1). Song minimum frequency varies spatially with environmental noise levels in this species (Derryberry et al., 2016; Luther, Phillips, & Derryberry, 2016). One mechanism to explain this correlation is that the song phenotype has changed over generations. Our previous work suggests that songs in this population have increased in minimum frequency over thirty years, consistent with increases in noise levels in San Francisco, CA over the same period of time (Luther & Baptista, 2010; Luther & Derryberry, 2012). This change in the song phenotype over time may be due to genetic changes or to ontogenetic adjustments, although the latter mechanism is more likely. A nonmutually exclusive explanation is that white‐crowned sparrow males shift song minimum frequency in immediate response to changes in noise levels. In this study, we examined the hypothesis that this species shows immediate flexibility in song minimum frequency. Using an observational approach, we addressed our hypothesis relative to noise levels taken over different periods of time: instantaneous (just prior to the song produced), bout level (during a song bout), and territory level (across song bouts for an individual bird). As vocal amplitude and sound frequency can be coupled in bird vocalizations (Beckers, Suthers, & ten Cate, 2003), we also asked whether song amplitude varied with noise levels and if song amplitude and song minimum frequency covaried. Given the logistical difficulties of obtaining song amplitude data on wild, free‐living birds, this was an exploratory, observational study to collect preliminary data to address this hypothesis before engaging in a larger scale, experimental study. Because white‐crowned sparrow males produce only one song type and sing with high stereotypy, such that one rendition of a song is very similar to the next, we did not predict that song minimum frequency would vary with noise levels, at least in immediate response to changes in noise levels. However, we did predict that song amplitude would vary with noise levels, as we have observed natural variation in song amplitude in this species. We also predicted that song amplitude and song minimum frequency might covary to some degree, as singing songs at higher frequency could allow for singing at a higher amplitude (Nemeth & Brumm, 2010).

## 2 METHODS

### 2.1 Study location and field recordings

We recorded 17 territorial white‐crowned sparrow (WCSP) males between 5:51 a.m. and 1:19 p.m. during the breeding season from June 11–19 2015. Male WCSP are more active in song production in the morning and during the breeding season. We opportunistically visited sites, walked in a randomly selected direction across sites until locating singing males. We then recorded the nearest male actively singing from an exposed perch at the time. All recordings were of spontaneous song (i.e., not elicited though playback). These focal males held territories in rural areas in Point Reyes National Seashore, including Commonweal and Abbott's Lagoon, or in urban parks in San Francisco, California, including Baker's Beach and Land's End. In this species, males holding territories within a location produce the same song type and locations tend to have different song types, known as “dialects” (Marler & Tamura, 1962). We sampled different dialects, and from both urban and rural areas, to broaden the interpretability of our findings.

Males were recorded using a Model 831 Larson Davis sound level meter ½ inch prepolarized omni‐directional microphone and calibrated preamplifier (Larson Davis, Depew, New York, USA). The sound level meter was stabilized and attached to a monopod. A shockmount was fitted to the monopod to reduce body movement noise. The sound level meter recorded sound pressure levels every 100 ms, and the detector type was set to fast. Sound pressure levels were A‐weighted LA_eq_. The LA_eq_ is the equivalent continuous sound that would contain the same sound energy as a noise that varies over time. An A weighting filter, which is an international standard, covers the frequency range of 20–20 kHz but the shape approximates the frequency sensitivity of the human ear, and to a certain extent the avian ear (Dooling & Popper, 2007). We also took 1/3‐octave band levels that were Z‐weighted L_eq_. A Z weighting filter is a flat frequency response. A ProStaff 3 6 × 21 Laser Rangefinder was used to measure the distance between the microphone and the bird when the distance was >10 m; otherwise, the distance was measured using a metric tape measure. The recording distance, bird orientation, and head movement were noted. Wind noise was minimized with a 3.5‐inch‐diameter windscreen. Wind speed was monitored with a handheld Kestrel 4,000 weather station to ensure songs were recorded at <1 m/s wind speed conditions.

### 2.2 Noise level data

**Figure 2:**
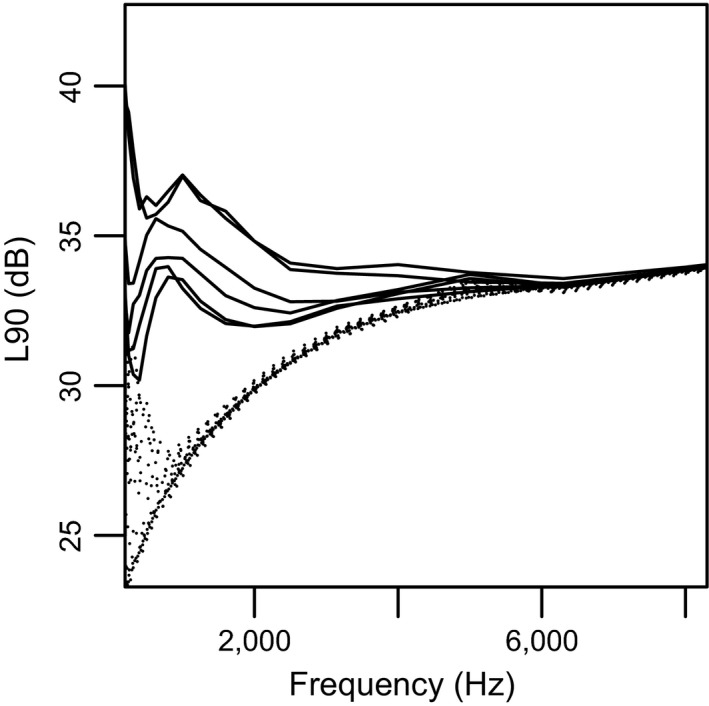
Spectral profile of background noise for every song bout sampled (L90 1/3 octave band levels). Background noise on territories within San Francisco city limits is indicated with solid lines and outside city limits with dotted lines

We used three measures of noise levels: (1) average LA_eq_ for ten seconds immediately prior to a song (hereafter, “instantaneous”), (2) the LAF_90_ for a song bout (hereafter, “bout background”), and (3) the median value of the LA_eq_ for all recordings on a given territory (hereafter, “territory”). The value LAF_90_ is the sound level exceeded 90% of the time and is a measure of background noise. We plot spectrums of the background noise levels during each song bout sampled in Figure 2 to illustrate variation in noise events across territories. We use median LA_eq_ instead of average LA_eq_ for territories, as median values capture information about noise levels measured over longer periods of time (here, over hours or days) and bigger areas. “Instantaneous” and “bout background” capture different levels of information because instantaneous measures are capped at 10 s, whereas song bouts are typically 2–3 min long and can be up to 10 min long. Calculations for instantaneous and median LA_eq_ are found in Equations 1 and 2 in the Supplementary Materials. The sound level meter provided the LAF_90_ values for a song bout. Noise levels are reported as dB(A) SPL (hereafter, “dB”) because this is a commonly accepted weighting used for reporting noise levels and effects on birds (Dooling & Popper, 2007). All reported sound pressure levels are in absolute units of dB reference 20 μPa.

### 2.3 Song minimum frequency

We measured the minimum frequency of each song (*n* = 75 songs, 17 birds) at −36 dB relative to the peak amplitude frequency from spectrograms (256 pt transform, frequency resolution: 97.7 Hz, 10.2 ms time resolution) in SIGNAL 5 (Beeman, 1999).

### 2.4 Song amplitude data

We used a subset of recorded songs to estimate song sound level (*n* = 28 songs, 16 birds). The amplitude of recorded songs was measured only when objects, such as tree branches or leaves, did not obstruct sound propagation and the bird was perched at the same height as the microphone. The recordings were also only considered measurable when a bird sang without lateral head movement and directly faced the microphone. The position and head movement of the bird was observed using Alpen Wing 8 × 42 binoculars.

We calculated song amplitude following methods described in Dooling and Popper (2007) and in Blickley and Patricelli (2012). We extracted focal songs (hereafter, “song segments”) and associated sound level meter data. Within the recording containing a focal song, we also located a segment of recording that did not contain a song (hereafter, “noise segments”) as close as possible in time to the focal song. We captured as long a segment of noise as possible, and noise segments ranged in length between 3 and 15 s. For the noise segments, we determined the noise level per second for each 1/3‐octave band from the sound level meter data. We used those bands in which WCSP songs occur (band center frequencies: 2,000–8,000 Hz). We then calculated an average noise level for the noise segment per band (using Equation 3). We next calculated the sound exposure level (SEL) per band for song segments (using Equation 4). We then removed from each band of song segments the amount of noise due to background noise from that band (as estimated from the noise segments) (see Equation 5). We calculated the SEL due to the song by summing across the bands (see Equation 6). We then corrected for distance of the recordist from the bird to determine the SEL_eq_ at 1 m from the bird (see Equation 7) (Marten & Marler, 1977). Song amplitude levels are reported as dB(Z) SPL (hereafter, “dB”) instead of dB(A) SPL as for estimates of noise levels, because we only included sound in octave bands that birds can hear. All equations are reported in Supplementary Materials.

### 2.5 Statistical analyses

Our samples included 77 songs from 17 males for models of song minimum frequency and 32 songs from the same 17 males for models of song amplitude (Table 1). To test our hypotheses, we used mixed‐effect general linear models and an information theoretic approach (Burnham & Anderson, 2002). We built models of the song trait (either song amplitude or song minimum frequency) as a function of noise level and selected the best descriptive models based on how well they fit the data using AIC_c_ (Akaike's Information Criterion) (Akaike, 1973) corrected for small sample size). The noise level variable differed among models. To test whether birds vary their songs immediately with changes in noise level, we included instantaneous noise level. To test whether birds vary their songs eventually with changes in noise level, we included bout background noise levels. For example, a loud event might occur at the start of a song bout, but the bird does not adjust its song until later in the song bout. For this reason, we might expect a stronger correlation of song features with song bout noise levels than with instantaneous noise levels. To test whether song features vary with noise levels measured over longer periods of time (over hours or days), we included territory noise levels. As the researcher's proximity to the bird varied among the recordings, we included distance from the recordist. We included both additive and interaction terms among measures of noise levels. We also tested whether song minimum frequency covaries with song amplitude by testing the fit of song amplitude against a null model.

**Table 1:** Sampling of males based on site type (urban/rural) and location. Number of songs (*n*) sampled per male for use in analyses of immediate flexibility in song minimum frequency (SMF) and in song amplitude (SA)

Site	Location	Bird	*n* (SMF)	*n* (SA)
Rural	Commonweal	Male 1	1	1
		Male 2	4	4
		Male 3	2	1
		Male 4	2	2
		Male 5	5	2
	Abbott's Lagoon	Male 6	3	1
		Male 7	4	1
		Male 8	16	8
		Male 9	5	2
		Male 10	7	3
		Male 11	3	1
Urban	Baker's Beach	Male 12	4	1
	Land's End	Male 13	3	1
		Male 14	3	1
		Male 15	3	1
		Male 16	7	1
		Male 17	5	1

Our previous work in this system indicated quadratic relationships between song features and noise levels (Derryberry et al., 2016). Therefore, we included quadratic terms of noise levels (e.g., instantaneous^2^) in our models. We also tested the fit of models with additive and interactive effects of different noise levels. Bird was included as a random effect in all models. In our models for song minimum frequency, we also included a random effect dependent on the background noise level and conditioned on each bird to account for the different noise events that birds could experience. Immediate signaling flexibility can be attributable to differences in the acoustic environment and/or prior experience of individual birds (Lazerte, Slabbekoorn, & Otter, 2016). Model selection using AIC_c_ values revealed which of the different combinations of fixed effects best explains song amplitude. In each model, the residual plots were inspected for heteroscedasticity and non‐normality. Analyses were run using R studio (R‐Core‐Team, 2016), foreach package (ANALYTICS, 2011), readexl package (Wickham, 2016), nlme package (Pinheiro, Bates, Debroy, & Sarkar, 2015), and the AICcmodavg package (Mazerolle, 2015). Scripts for all analyses can be found in “AmpFreqAnalysis.R” and associated data files are “AmbientNoiseMeasures.csv,” “SongAmplitude2015.csv,” and “MinFreqResults.csv.”

For each song trait, we report the top model, models within at least 2 ΔAICc, and the null model. We present effect sizes (β) ± standard errors (*SE*) for the top model, parameter total weights, and measures of support for models, including the weight of the model of interest (*w*_i_) and the evidence ratio (ER = *w*_i_/w_null model_), which is interpreted as the probability that the model of interest is the best model in the set as compared to an appropriate null model.

## 3 RESULTS

### 3.1 Song minimum frequency does not vary with ambient noise levels

We found no support for any of the three measures of noise level (instantaneous, bout background or territory) explaining variation in song minimum frequency (top model: constant model; ER = 1; Table 2). For all three noise terms, the relative importance (i.e., total weight) was <0.4. Thus, changes in noise levels over short time periods do not explain variation in minimum frequency between songs.

**Table 2:** Rank of models that describe song minimum frequency relationship with noise levels

Model	*K*	AICc	ΔAICc	*w*_i_
Intercept only (null) model	3	946.77	0	0.23
Bout Background	4	948.20	1.43	0.11
Territory	4	948.62	1.85	0.09
Bout Background + Territory	5	948.74	1.97	0.09

Top model and models within 2 ΔAICc are shown; *K*, number of parameters in model; AICc, Akaike information criterion with a correction for finite sample sizes; ΔAICc, difference between each model's AICc and that of the best model; *w*_i_, model weight.

### 3.2 Song amplitude varies with instantaneous noise levels

**Figure 3:**
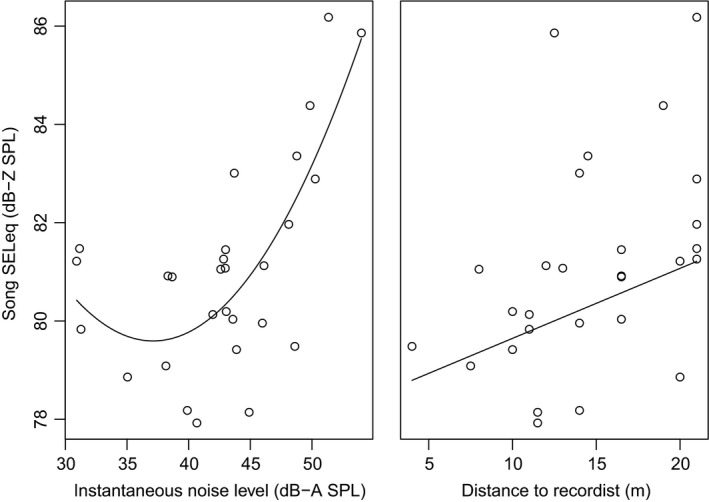
Song amplitude increases with instantaneous noise levels and distance to recordist. Instantaneous noise levels were taken 10‐s prior to song production. In general, birds sing louder in response to immediate increases in noise level. Birds also sing louder the greater the distance from the recordist

As predicted, song amplitude was positively related to noise levels. The top ranked model included instantaneous and instantaneous^2^ as well as a distance term and received 53% of the model weight with a high evidence ratio (ER > 17,086; Table 3). Song amplitude increased over the range of instantaneous noise levels as a result of a quadratic relationship (instantaneous: β = −1.6 dB/dB ± 0.48; instantaneous^2^: β = 0.022 dB/dB^2^ ± 0.01). Below roughly 40 dB of ambient noise, males do not appear to adjust their song amplitude. However, above 48 dB of ambient noise, an increase of 12 dB (i.e., noise quadrupling) results in song amplitude increasing by 8.7 dB (i.e., tripling in loudness) (see Figure 3). Birds also sang louder the greater the distance from the recordist (β = 0.14 dB/m ± 0.05 *SE*), such that birds sang 12% louder (i.e., ~1 dB) for each additional seven meters between them and the recordist. The distance parameter has strong support for inclusion in a model describing variation in song amplitude, as it has a total weight of 0.82 across all models. Instantaneous noise level had a total weight of 0.94 and instantaneous^2^ of 1, providing strong support for these parameters explaining variation in song amplitude. There was low support for inclusion of any other terms in the model (parameter total weights <0.2), providing evidence that song amplitude varies primarily with instantaneous noise levels and with distance from a recordist.

**Table 3:** Rank of models that describe song amplitude relationship with noise levels

Model	*K*	AICc	ΔAICc	*w*_i_
Distance + Instantaneous + Instantaneous^2^	6	103.4	0	0.53
Distance + Instantaneous^2^ + Territory	7	106.7	3.3	0.1
Distance + Instantaneous + Instantaneous^2^ + Bout Background	7	107.0	3.6	0.09
Instantaneous + Instantaneous^2^	5	107.3	4	0.07
Intercept only (null) model	3	122.9	19.5	0

Top model and models within 4 ΔAICc and null model are shown; *K*, number of parameters in model; AICc, Akaike information criterion with a correction for finite sample sizes; ΔAICc, difference between each model's AICc and that of the best model; *w*_i_, model weight.

### 3.3 Song amplitude and song minimum frequency do not covary

We did not find evidence of covariation between song amplitude and song minimum frequency. Song amplitude did not explain variation in song minimum frequency better than a null model (Null AICc = 402.26; Song Amplitude AICc = 402.78).

## 4 DISCUSSION

**Figure 4:**
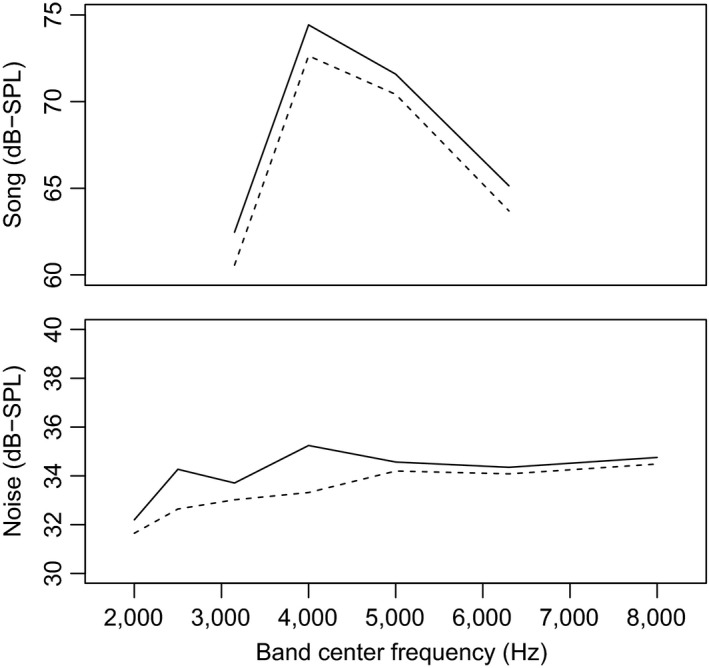
Song amplitude, but not song minimum frequency, shifts with changes in instantaneous noise levels. Top panel shows how energy is distributed across frequencies during song production for two songs drawn from the same song bout. The solid line illustrates the relatively louder song. Note that both songs show the same distribution of sound across frequencies, indicating no shift in song minimum frequency. Bottom panel shows the distribution of energy across frequencies for background noise from the 10‐s prior to production of each song in the top panel. The solid line illustrates the background noise for the relatively louder song. Note that it has more energy at lower frequencies

We find that song amplitude, but not song minimum frequency, changes in predicted directions with changes in background noise levels over a timescale of seconds (Figure 3). Specifically, when background noise levels increase, males immediately sing louder, as illustrated in Figure 4. We did not find a correlation between song amplitude and song minimum frequency in our dataset. Our results suggest that (1) males do not show flexibility in song minimum frequency in response to fluctuations in noise levels on an immediate time scale, (2) males do show immediate flexibility in song amplitude, and (3) that white‐crowned sparrows do not face a physiological constraint on singing louder at these frequencies. Together, these results show support for immediate flexibility in song amplitude as a behavioral response to the soundscape and indicate additional work is needed to examine other mechanisms, such as ontogenetic adjustments, to explain previously described correlations between song minimum frequency and environmental noise levels across territories and over generations in this species (Derryberry et al., 2016; Luther & Baptista, 2010).

### 4.1 No change in song minimum frequency with noise levels

We did not find evidence that white‐crowned sparrow males shift their song minimum frequencies when background noise levels vary in their environment. To place our results in context, previous observational studies find that some bird species vary in their degree of immediate flexibility in song minimum frequency such that some species produce vocalizations with higher minimum frequencies in areas with higher environmental noise levels (e.g., Hu & Cardoso, 2010) whereas others do not (Francis, Ortega, & Cruz, 2010; Ríos‐Chelén, Salaberria, Barbosa, C, & Gil, 2012; Ríos‐Chelén, Quirós‐Guerrero, et al., 2012). Most experimental studies that have reported measuring song minimum frequency have found that individuals shift minimum frequency higher when exposed to city‐like noise (Bermudez‐Cuamatzin, Rios‐Chelen, Gil, & Garcia, 2011; Gross, Pasinelli, & Kunc, 2010; Montague, Danek‐Gontard, & Kunc, 2012; Verzijden, Ripmeester, Ohms, Snelderwaard, & Slabbekoorn, 2010). However, a recent study found birds may need prior experience with noise in order to learn to adjust their vocalizations in ways that reduce masking (Lazerte et al., 2016). In cases supporting immediate flexibility, birds have been found to shift minimum frequency of individual notes within a song type (Bermudez‐Cuamatzin et al., 2011), sing a greater proportion of higher frequency notes (Ripmeester, Kok, van Rijssel, & Slabbekoorn, 2010; Ripmeester, Mulder, & Slabbekoorn, 2010), or switch to song types with higher minimum frequencies (Halfwerk & Slabbekoorn, 2009). In studies that do not find a shift in minimum frequency, the species in question can show flexibility in another feature of song that reduces masking, such as increasing song length (Ríos‐Chelén, Quirós‐Guerrero, et al., 2012) or potentially song amplitude (Brumm & Zollinger, 2013), although few studies of birds measure both features of song.

Our motivating question in this study was to understand whether short‐term adjustments in song minimum frequency could explain why this feature of song varies with background noise levels in this species. Our previous work documented variation in song minimum frequency at a number of temporal and spatial scales. Song minimum frequency in this species has changed across generations (approximately 30 years) in association with changes in the transmission properties of its environment (Derryberry, 2009) and with changes in background noise levels in its soundscape (Luther & Baptista, 2010; Luther & Derryberry, 2012). Further, we found evidence of spatial variation in song minimum frequency across soundscapes consistent with the acoustic adaptation hypothesis (Derryberry et al., 2016). Findings in this study suggest that these temporal and spatial patterns of variation are likely not the result of immediate flexibility in song minimum frequency. However, we acknowledge that this was an exploratory and observational study. To examine the immediate flexibility hypothesis further will require measuring behavioral response to experimental manipulations of noise exposure.

### 4.2 Increase in song amplitude with increasing noise levels

White‐crowned sparrow males sing louder when noise levels in their environment increase. The noise level immediately preceding a given song predicted variation in song amplitude, suggesting that males can respond immediately to changes in background noise levels (Figures 3 and 4). We found that males sampled over background noise levels between 30 and 54 dB(A) SPL produced songs between 77 and 86 dB(Z) SPL. This relationship between song amplitude and background noise levels had a quadratic term, such that males appear to adjust their song amplitude only above a certain noise threshold. When background noise is below 40 dB, males do not change their song amplitude, but above about 48 dB, males will triple their sound pressure level (8.7 dB increase) for every 12 dB increase in background noise level. Our pattern of results is consistent with those found for the short list of species in which a correlation between vocal amplitude and background noise level has been demonstrated in the wild. These species include Common Nightingales (*Luscinia megarhynchos*) (Brumm, 2004), male Blue‐throated Hummingbirds (*Lampornis clemenciae*) (Pytte, Rusch, & Ficken, 2003), Noisy Miners (*Manorina melanocephala*) (Lowery, Lill, & Wong, 2012), and Eastern Bluebirds (*Sialia sialis*) (Kight & Swaddle, 2015). In these studies, vocal amplitudes were compared to environmental noise levels close to the time of the vocalization (comparable to our estimates of “instantaneous noise levels”). In nightingales, males sampled on territories that had a mean noise level between 40 and 64 dB(A) produced songs between 75 and 92 dB(A), such that males sang approximately 9.5 dB(A) louder for every 12 dB increase in environmental noise levels (β = 0.794) (Brumm, 2004). In noisy miners, individuals were recorded in localities that ranged from ~41 to 74 dB and produced calls between 65 and 100 dB, such that males sang approximately 10 dB louder for every 12 dB increase in environmental noise levels (inferred from figure 2 in Lowery et al., 2012). We cannot compare our findings directly to those reported for Eastern Bluebirds, as that study reduced dimensionality of song features and so reports results for principal components and not individual song features (Kight & Swaddle, 2015). Overall, our findings of changes in vocal amplitude in response to instantaneous changes in background noise levels are comparable to those for other passerines, although it appears white‐crowned sparrows may show a lower responsiveness in terms of vocal intensity.

Differences in song amplitude observed among the white‐crowned sparrow males are likely the result of the Lombard effect (Lombard, 1911), or noise‐dependent regulation of vocal amplitude. The Lombard effect is taxonomically widespread (Brumm & Zollinger, 2011). Experimental studies of the Lombard effect in eight species of birds across six families have found evidence that birds change the amplitude of their vocalizations in response to changes in ambient noise levels (reviewed in Brumm & Zollinger, 2013), although it is clear that noise alone is not enough to induce an increase in vocal amplitude; spectral overlap between the vocal signal and noise is necessary (Manabe, Sadr, & Dooling, 1998). Similar field studies demonstrating an association between song amplitude and ambient noise levels suggest that the Lombard effect is the best explanation of this correlation (Brumm, 2004). However, conclusive support for the Lombard effect in white‐crowned sparrows requires experimental tests.

### 4.3 Song amplitude and frequency do not covary

We did not find support for our hypothesis that song minimum frequency and song amplitude would covary, suggesting that there is not a constraint on song amplitude at the given frequencies examined here for white‐crowned sparrows. We expected an association between these song traits because frequency and amplitude are often coupled in vocal production in birds (Amador & Margoliash, 2013; Beckers et al., 2003; Nelson, B. S., 2000). The idea is that when amplitude increases due to the Lombard effect, there is a passive increase in frequency, which has been shown in mammals (Lu & Cooke, 2008). However, an experimental study of this effect in bats found that shifts in call frequency and in amplitude within the Lombard effect are independent, suggesting different neural mechanisms underlying these changes (Hage et al., 2013). There are very few studies that examine covariation in vocal amplitude and frequency in birds, at least in the context of ambient noise levels. Comparing our study to these few suggests that our results are not atypical. At least three laboratory‐based experimental studies of the Lombard effect have found shifts in both vocal amplitude and frequency in birds (Schuster et al. 2012, Manabe et al., 1998; Omanski and Dooling 2009). However, one study on Tree Swallows (*Tachycineta bicolor*) found a shift in vocal amplitude but not frequency in the laboratory, although both vocal features shifted in the field (Leonard & Horn, 2005). Noisy Miners appear to increase vocal amplitude but not vocal frequency (Hu & Cardoso, 2010; Lowery et al., 2012), whereas Eastern Bluebirds adjust both amplitude and frequency (Kight & Swaddle, 2015) in observational field studies. We know of no studies that have measured both features of song in field studies of vocal responses to manipulations of noise exposure. The lack of such studies reflects the logistical difficulties of measuring song amplitude in the field, and methodological biases in measuring song minimum frequency (Zollinger, Podos, Nemeth, Goller, & Brumm, 2012). Laboratory‐based experimental studies are needed to test whether noise induced shifts in song amplitude also result in shifts in song minimum frequency in white‐crowned sparrows.

### 4.4 Distance from recordist affects song amplitude

We found an effect of the recordist on song amplitude. We found that birds sang more softly the closer the person holding the recording equipment stood. One possible reason for this behavior is that reducing song amplitude may decrease detectability. Given that white‐crowned sparrows often sing at perch heights comparable to human height, this species may categorize humans as a potential threat. A human standing closer to a bird watching their behavior may therefore be perceived as a higher threat leading to behavior that reduces detection. Although the effect of an observer is well known, such that territorial playback experiments often take place with observers 20 m + from the playback speaker (McGregor et al., 1992), we do not know of any study that accounts for the effect of a recordist on behavior from analyses of song recordings. Our findings suggest that future studies should include this variable in analysis of song amplitude, particularly of species that sing from relatively low perches. A straightforward means to include this information is distance from the recordist, which can be collected using a range finder device if the location of the bird can be determined.

### 4.5 Short‐term adjustments of behavior in response to variable soundscapes

If we infer process from these patterns, our findings suggest that the short‐term, immediate reaction to noise events in white‐crowned sparrows is to sing louder, not higher. Flexibility in terms of song amplitude appears to be on the order of seconds. In support of this generalization, we measured individual birds singing 2–6 dB louder (6 dB is a doubling of sound pressure levels) in response to changes in noise levels just prior to a song. In contrast, males did not adjust song minimum frequency on the same time scale. Together, our findings suggest more flexibility in adjustment of song amplitude than in adjustment of song minimum frequency in this species.

Another interpretation of our results is that individuals are adjusting their song in the most advantageous manner possible. In other words, males may be able to shift song minimum frequency, but do not do so in response to changes in noise levels if shifting song minimum frequency does not reduce masking. Nemeth and Brumm (2010) demonstrated in other bird species that adjustment of song amplitude increases transmission distance more in the face of masking than does adjustment of song minimum frequency. In addition, louder songs tend to elicit a stronger response from intended receivers (Ritschard, Riebel, & Brumm, 2010; Searcy, 1996), but less so with adjustments of minimum frequency (Luther, Danner, Danner, Gentry, & Derryberry, 2016). We did not calculate how adjustment in song amplitude or song minimum frequency would effect transmission distance in this species, but it is possible that adjusting song amplitude is more effective than adjusting song minimum frequency, at least in this soundscape for this species.

## 5 CONCLUSIONS

White‐crowned sparrows show short‐term adjustments in song amplitude in immediate response to changes in background noise levels, but do not show the same flexibility in song minimum frequency. Most other species examined show immediate flexibility in song amplitude, but species vary in flexibility in song minimum frequency. Further, we find no evidence of constraints at these song frequencies on adjusting song amplitude, as these two features do not covary, although we note that white‐crowned sparrows show smaller adjustments in song amplitude than other species examined to date. We place our findings in the context of previous research on this species and suggest that immediate flexibility cannot explain spatial or temporal variation in song minimum frequency with background noise levels. However, experimental studies are needed to test this hypothesis further. We highlight the need to examine multiple mechanisms to explain variation in vocal behavior with natural and anthropogenic sources of noise—including genetic change, adjustments during development, and immediate flexibility—as there are no species in which all of these mechanisms have been addressed in order to assess their relative role in shaping vocal behavior.

## CONFLICT OF INTEREST

None declared.

## AUTHOR CONTRIBUTIONS

EPD, RMD, JED, and DAL conceived the study. EPD wrote the initial manuscript. KEG recorded the songs. EPD, KEG, JNP, and GED conducted analyses. All authors contributed to editing and revising the manuscript.
